# Supplementary material for: MetaRibo-Seq measures translation in microbiomes
Source: Nat Commun. 2020 Jun 29;11:3268. doi: 10.1038/s41467-020-17081-z (PMC7324362; doi:10.1038/s41467-020-17081-z)
Supplement: Supplementary file 10 — Supplementary Data 7 [file 41467_2020_17081_MOESM10_ESM.zip › File2/Confidence_High_Taxonomy/305892_out.krona.html]

Javascript must be enabled to view this page.

members
magnitude
magnitudeUnassigned
count
unassigned
taxon
rank

305892\_out

55

55
2
superkingdom

201174
phylum
55

1760
class
55

order
85004
55

55
31953
family

55
genus

SRS012273\_contig\_number\_11611
1
1678


SRS101376\_contig\_number\_7975
1683
species
1

4

SRS014683\_contig\_number\_10500SRS042966\_contig\_number\_contig-100\_42.80716SRS050422\_contig\_number\_19072SRS147557\_contig\_number\_contig-100\_7002.137271
species
1681

216816
5

SRS018427\_contig\_number\_contig-100\_35494.35494SRS022071\_contig\_number\_33985SRS098717\_contig\_number\_20606SRS1041038\_contig\_number\_5925SRS146812\_contig\_number\_49705
species
10

5

SRS017745\_contig\_number\_2356SRS1041091\_contig\_number\_16597SRS143466\_contig\_number\_28460SRS147039\_contig\_number\_3891SRS147977\_contig\_number\_2098
subspecies
1679

29
species

SRS011239\_contig\_number\_contig-100\_6591.140149SRS012969\_contig\_number\_contig-100\_6379.91971SRS013098\_contig\_number\_38652SRS015431\_contig\_number\_21779SRS015578\_contig\_number\_27516SRS017103\_contig\_number\_41555SRS018541\_contig\_number\_2202SRS019068\_contig\_number\_106239SRS020328\_contig\_number\_contig-100\_2806.187130SRS023526\_contig\_number\_contig-100\_13872.164782SRS023715\_contig\_number\_contig-100\_2124.158486SRS024132\_contig\_number\_contig-100\_3391.236844SRS053356\_contig\_number\_25645SRS056273\_contig\_number\_11023SRS064276\_contig\_number\_36198SRS075878\_contig\_number\_19448SRS077454\_contig\_number\_19828SRS1041033\_contig\_number\_20294SRS1041039\_contig\_number\_contig-100\_5166.123604SRS1041133\_contig\_number\_25253SRS1055056\_contig\_number\_11052SRS143523\_contig\_number\_9375SRS144135\_contig\_number\_22174SRS148159\_contig\_number\_29671SRS148196\_contig\_number\_40366SRS150029\_contig\_number\_21165SRS892882\_contig\_number\_4590SRS893366\_contig\_number\_contig-100\_2159.139432SRS971275\_contig\_number\_37343
1680

4
1685

SRS024331\_contig\_number\_24286SRS075975\_contig\_number\_34SRS075977\_contig\_number\_contig-100\_189.189SRS075979\_contig\_number\_contig-100\_27.27
species


SRS1055043\_contig\_number\_contig-100\_1207.128405
28026
species
1

5

SRS018313\_contig\_number\_1822SRS051610\_contig\_number\_1487SRS074670\_contig\_number\_2847SRS104912\_contig\_number\_5003
species
4
1686


SRS015890\_contig\_number\_13249
630129
subspecies
1
